# Muscle Matters: Dietary Supplement Use and Sarcopenia Risk

**DOI:** 10.3390/geriatrics11040092

**Published:** 2026-07-21

**Authors:** Francesco Saverio Ragusa, Anna Licata, Pascal R. Titone, Alessandro D’Aleo, Gerlando Speziale, Laura Vernuccio, Maurizio Soresi, Giovanna Di Bella, Lydia Giannitrapani, Nicola Veronese, Ligia J. Dominguez, Mario Barbagallo

**Affiliations:** 1 Geriatric Unit, Via Del Vespro 129, 90127 Palermo, Italy; 2 Internal Medicine & Hepatology Unit, AOUP “P. Giaccone”, Department of Health Promotion Sciences, Maternal and Infant Care, Internal Medicine and Medical Specialties, PROMISE, School of Medicine, University of Palermo, Piazza Delle Cliniche 2, 90127 Palermo, Italy; 3 Faculty of Medicine, Saint Camillus International University of Health Sciences, Via Di Sant’Alessandro 8, 00131 Rome, Italy; 4 Department of Psychology, Educational Science and Human Movement, University of Palermo, Via Pascoli 6, 90144 Palermo, Italy

**Keywords:** sarcopenia risk, dietary supplements, older people

## Abstract

**Background:** The association between dietary supplement use and risk of sarcopenia in older adults remains poorly investigated. The primary objective of this study was to investigate the association between risk of sarcopenia and supplement use in older adults. **Methods:** A cross-sectional study was conducted to analyze the association between supplement use and risk of sarcopenia, assessed by the SARC-F questionnaire, in older outpatients. The associations between risk of sarcopenia and dietary supplement use were examined through logistic regression models. **Results:** A total of 162 participants were included in this study. Among supplement users (92 patients), 55.4% were identified as being at risk of sarcopenia (SARC-F ≥ 4). Regarding SARC-F components, dietary supplement users exhibited significantly higher median scores for walking assistance (median 1 vs. 0, *p* = 0.004) and rising from a chair (median 1 vs. 0, *p* = 0.04) compared to non-users. Individuals at risk were significantly more likely to use dietary supplements compared with those not at risk (OR 1.98, 95% CI 1. 05–3.76; *p* = 0.034), and this association remained significant after adjustment for confounders, such as sex, age, study, and marital status (OR 2.35, 95% CI 1.21–4.72; *p* = 0.013). Cognitive decline was the most frequently reported reason for supplement use in both groups. **Conclusions:** Dietary supplement use is highly prevalent among older adults and independently associated with sarcopenia risk, particularly tracking with functional limitations. Rather than a causal factor, supplementation may serve as a surrogate indicator of overall clinical vulnerability. These findings underscore the importance of assessing muscle health in older supplement users.

## 1. Introduction

Sarcopenia, defined as age-related loss of muscle, involving reductions in appendicular muscle mass, muscle strength, and/or physical performance [1], is prevalent in older adults, with roughly 10% of individuals over 60 affected, regardless of sex [2]. Sarcopenia is a major contributor to disability and frailty, with shared underlying factors including age-related chronic inflammation, alterations in body composition, and hormonal imbalances [3]. It has also been associated with an increased risk of cognitive impairment in the older population [4]. A valid and quick screening tool to identify older adults at risk of sarcopenia is SARC-F. It evaluates muscle strength, physical performance, and functional abilities such as walking speed, chair rise, climbing stairs, and history of falls [5].

In high-income countries, a substantial majority of older adults report regular use of dietary supplements, and a notable proportion of older individuals in other regions do so as well [6,7]. Supplement consumption in this population is often habitual and long-term, with many individuals taking multiple products concurrently, including vitamins, minerals, herbal preparations, and other nutraceuticals [8,9]. This widespread and frequently combined use reflects growing interest in self-directed health management and disease prevention among aging populations [10].

Supplements have been shown to exert beneficial effects on walking speed and muscle strength in older adults [11], i.e., the two key components associated with the risk and progression of sarcopenia. On the other hand, no studies were identified that directly investigated the use of supplements in patients already at risk of sarcopenia. Most existing research has approached the issue from the opposite perspective, examining how supplement use might reduce the risk of developing sarcopenia. For example, a Chinese study involving 7864 participants found that higher dietary intake of supplements was significantly associated with a lower risk of sarcopenia [12]. A British systematic review and meta-analysis, after screening 28 studies on nutritional supplements, showed improvements in skeletal muscle mass index (+29%) and total fat mass (+21%) in those who took supplements [13]. On the other hand, there is also current evidence regarding the efficacy of these dietary interventions, which remains highly inconsistent. A recent meta-analysis of 35 studies and 6628 older participants demonstrated that vitamin D supplementation had a minimal effect on sarcopenia parameters, showing no significant impact on appendicular skeletal muscle mass, handgrip strength, or physical performance via the Timed Up and Go test [14]. Nevertheless, nutritional deficits and use of dietary supplements remain critical in musculoskeletal health. For instance, data from an Italian study on 2756 older adults showed that low vitamin D levels significantly increase the odds of osteoarthritis (by 26%) and joint pain (by 18%) [15]. This interplay between nutrient levels and musculoskeletal degeneration further underscores the complexity of this topic and justifies the need to investigate whether individuals at higher risk of sarcopenia are more likely to use dietary supplements.

The primary objective of this study was to investigate the association between risk of sarcopenia and supplement use in older adults. Addressing a meaningful gap in the current literature, we focused specifically on individuals already at risk of sarcopenia. Clarifying supplement patterns in this vulnerable subgroup is clinically essential, as it helps determine whether supplementation represents an unguided reactive strategy to initial physical decline or a surrogate indicator of overall clinical frailty. Specifically, we aimed to assess whether individuals at higher risk of sarcopenia were more likely to use dietary supplements, taking into account potential confounding factors such as sex, age, marital status, educational level, and smoking.

## 2. Materials and Methods

### 2.1. Patients Population

A cross-sectional study was carried out between 1 February 2024 and 1 February 2025 at the Department of Internal Medicine and Geriatrics of the University of Palermo, Italy. The study population consisted of men and women older than 60 years who attended outpatient clinics dedicated to the management of cognitive disorders and osteoporosis. The study protocol was approved by the Palermo 1 Ethics Committee during the meeting held on 12 December 2023 (protocol no. 07/2023).

Written informed consent was obtained from all participants. Only patients with a Mini-Mental State Examination score > 24 were eligible for enrolment. This threshold guaranteed that all participants, including those reporting mild cognitive decline as a reason for supplement use. When participants were not fully able to understand the consent process, the study team reviewed the information with a caregiver to ensure adequate comprehension. Individuals younger than 60 years were excluded from the study.

### 2.2. Supplements

We defined supplements according to European Directive 2002/46/EC [16] as concentrated sources of nutrients or other substances with a nutritional or physiological effect. Supplement use was assessed during the interview in the outpatient visit. Patients were asked about their current, regular use (defined as daily or weekly intake for at least the past 3 months) rather than occasional consumption.

### 2.3. Questionnaires

Data regarding dietary supplement use were collected during the clinical interview using a structured questionnaire about use and consumption; details are provided in Supplementary Table S1. It is worth noting that all participants identified as supplement users were already on active, ongoing therapy at the time of enrollment. No dietary supplements were prescribed or modified during the specific outpatient visit in which the study questionnaires were administered, ensuring that supplement use reflected a pre-existing clinical habit rather than an immediate consequence of the current evaluation.

The SARC-F questionnaire is a simple, validated screening tool for sarcopenia in older adults. It was entirely interviewer-administered by trained medical staff during face-to-face clinical evaluations. It evaluates five domains: strength, assistance with walking, rising from a chair, climbing stairs, and history of falls. Total scores range from 0 to 10, with scores ≥ 4 indicating risk of sarcopenia (Supplementary Table S2).

### 2.4. Statistical Analyses

Continuous variables were summarized using means and standard deviations, whereas categorical variables were described using frequencies and percentages. Baseline characteristics were compared according to supplement use using chi-square tests for categorical variables or Fisher’s exact test when expected cell counts were below five.

Variables derived from the SARC-F questionnaire, including its individual domain scores and the total number of falls, were treated as ordinal and non-parametric data. Consequently, these variables were expressed as medians and interquartile ranges (IQR). The statistical comparison between dietary supplement users and non-users for these categorical/ordinal outcomes was performed using the non-parametric Wilcoxon rank-sum test (also known as the Mann–Whitney U test).

Associations between risk of sarcopenia (SARC-F ≥ 4) and supplement use were evaluated by calculating odds ratios (ORs) with 95% confidence intervals (CIs). Both univariable (unadjusted) and multivariable (adjusted) logistic regression analyses were performed. The multivariable model was adjusted for potential confounding factors identified a priori, including age, sex, marital status, educational level, and smoking status, to isolate the independent association between sarcopenia risk and dietary supplement use. Participants without sarcopenia risk served as reference groups. All tests were two-sided, and statistical significance was defined as *p* < 0.05. Analyses were performed using RStudio (Version 2024.12.1+563).

## 3. Results

### 3.1. Descriptive Characteristics of Patients

Table 1 presents the descriptive characteristics of the study population stratified by supplement use. Among the 162 participants, 92 (56.8%) reported using dietary supplements. The mean age was similar between users and non-users (75.1 ± 6.7 vs. 74.6 ± 8.0 years, *p* = 0.67). Supplement users had a higher prevalence of being at risk of sarcopenia (SARC-F ≥ 4) compared with non-users (55.4% vs. 38.6%, *p* = 0.04). No significant differences were observed between the two groups regarding sex distribution, marital status, educational level, residence, smoking, alcohol consumption, or reported healthy habits.

Regarding the SARC-F components, functional measures showed that dietary supplement users exhibited significantly worse limitations compared to non-users in assistance in walking (median 1, IQR [0–2] vs. median 0, IQR [0–1]; *p* = 0.004) and rising from a chair (median 1, IQR [0–1] vs. median 0, IQR [0–1]; *p* = 0.04). Conversely, no statistically significant differences were observed between the two groups for SARC-F muscle strength (*p* = 0.25), stair climbing (*p* = 0.18), and the number of falls (*p* = 0.75). Among supplement users, the majority reported benefits (66.3%), experienced no side effects (95.7%), and had initiated supplementation mainly under specialist guidance (83.7%), with general practitioners informed in most cases (91.3%). Nutritional status, assessed by MNA, did not differ significantly between groups, with roughly 44% malnourished, 28% at risk, and 27% having normal status (*p* = 0.71).

### 3.2. Association of Supplement Use with Sarcopenia Risk

Use of dietary supplements was more common among patients at risk of sarcopenia. In unadjusted analysis (Table 2, Model 1), people at risk of sarcopenia had an OR of 1.98 (95% CI 1.05–3.76; *p* = 0.034) of taking dietary supplements. After adjustment for sex, age, marital status, educational level, and smoking (Table 1, Model 2), the association remained significant (OR 2.35, 95% CI 1.21–4.72; *p* = 0.013), suggesting that patients at risk of sarcopenia were more than twice as likely to use dietary supplements compared with those not at risk.

### 3.3. Supplement Use by Sarcopenia Risk Status

**Figure 1:**
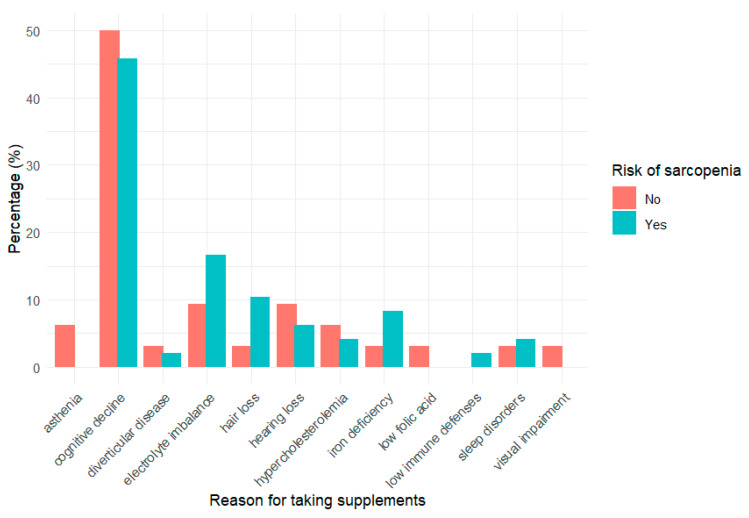
Reasons for supplement use by sarcopenia risk status.

Figure 1 illustrates the distribution of reasons for taking supplements according to sarcopenia risk. Cognitive impairment emerged as the primary driver for supplementation in both cohorts, showing a significantly higher prevalence among individuals at risk of sarcopenia (23.9% vs. 22.9%; *p* = 0.020). Conversely, other reported motivations, such as asthenia, electrolyte imbalances, and nutritional deficiencies, showed balanced distributions with no statistically significant differences between the two groups (all *p* > 0.05). Clinically, the vast majority of supplements were specialist-prescribed (83.7%), yielding a perceived positive benefit in two-thirds of the sample 66.3%). Side effects were reported in only 4.3% of participants. Regarding the specific types of products consumed by the study population, dietary supplements constituted the largest proportion, accounting for 46.59% of the total usage. This was followed by oligoelements (17.05%) and herbal products (14.77%). Vitamins represented 12.50% of the intake, while the remaining 9.09% was classified as other miscellaneous formulations.

Overall, these results suggest that patients at risk of sarcopenia have lower physical function and are more likely to use dietary supplements, with cognitive decline being the predominant motivation for supplementation.

## 4. Discussion

In our study, we investigated the association between use of supplements and risk of sarcopenia in older adults. Specifically, we sought to evaluate whether individuals at higher risk of sarcopenia were more likely to use dietary supplements, taking into consideration potential confounding factors such as sex, age, marital status, educational level, and smoking.

55.4% of the supplement users were identified as being at risk of sarcopenia (SARCF score ≥ 4), highlighting that this condition is relatively prevalent among older adults who use dietary supplements. This finding underscores the potential importance of monitoring muscle health in this population, as a substantial proportion of supplement users may already be experiencing early signs of muscle decline. Such a high prevalence also suggests that interventions aimed at preventing or mitigating sarcopenia could be particularly relevant for individuals who are proactive in taking supplements, emphasizing the need for targeted strategies to support musculoskeletal health.

Analyzing the individual components of the SARC-F, we found that dietary supplement users exhibited significantly worse distributions, with higher median scores for walking assistance and rising from a chair compared to non-users. Conversely, no significant differences were observed for the muscle strength component. Our results were confirmed by previous literature: supplements, particularly multi-nutrient or protein-containing supplements, have been shown to improve handgrip strength and chair-rise performance in older adults with frailty or sarcopenia, as shown by an Italian systematic review and meta-analysis of 32 RCTs for a total of 4137 older participants, where compared with placebo, multi-nutrient supplementation was associated with notable improvements in physical performance, including faster chair rise times [17]. Together, these data support the plausibility that older supplement users in your study would present poorer SARC-F component scores related to strength and functional mobility than non-users.

Interestingly, our data reveal that dietary supplement use was overwhelmingly driven by medical advice rather than self-medication, with 93.4% of users receiving recommendations from medical doctors. Given that supplement users scored significantly higher in negative SARC-F components (such as assistance in walking and rising from a chair), it is highly probable that these products were recommended by clinicians as a reactive strategy to manage early signs of physical frailty, sarcopenia risk, or co-existing conditions like cognitive decline.

In the present study, dietary supplement use exhibited a significant, independent association with an increased risk of sarcopenia. Although cross-sectional analyses are inherently susceptible to reverse causality, making it impossible to definitively establish the causal direction, several clinical characteristics of our cohort merit consideration. Data from clinical interviews suggested that supplement use predominantly reflected a long-standing therapeutic regimen rather than an immediate reactive strategy instituted during the current evaluation; notably, no new supplements were prescribed during the study visit.

Furthermore, the primary clinical indications driving supplementation in this sample were largely non-musculoskeletal, encompassing cognitive decline, electrolyte imbalances, alopecia, and visual impairment. While these underlying conditions do not directly stem from muscle loss, we cannot rule out that they represent unmeasured indicators of frailty. Therefore, rather than implying a direct causal link, these findings suggest that dietary supplement use may serve as an independent surrogate indicator of overall clinical vulnerability and risk of sarcopenia in older outpatients. Prospective longitudinal studies remain strictly necessary to distinguish whether a higher sarcopenia risk drives supplement use or if supplementation merely reflects underlying factors related to muscle decline. Most of the available research has taken the reverse approach, focusing instead on whether supplements use can help prevent the onset of sarcopenia: a Chinese systematic review and meta-analysis of eight studies involving 1204 participants found that dietary supplement interventions significantly improved body weight, muscle mass, muscle strength, and physical performance [18], but the number of studies included was too small and the follow-up was too short. The higher prevalence of supplement use among at-risk individuals may reflect increased health awareness, clinical recommendations, or participation in nutritional and rehabilitation programs. Additionally, supplements targeting muscle health may be perceived as beneficial in counteracting early functional decline, which could further explain their more frequent use in this population.

Individuals at risk of sarcopenia frequently exhibit insufficient intake of protein and key micronutrients, together with a phenomenon known as anabolic resistance [19], whereby skeletal muscle displays a blunted synthetic response to usual dietary protein stimuli. In this context, supplementation strategies providing high-quality protein and essential amino acids may help stimulate muscle protein synthesis, preserve or increase appendicular and total lean mass, enhance muscle strength and physical function and improve the immune system [20,21]. These effects appear to be more pronounced when nutritional interventions are combined with resistance training or structured exercise programs [22].

Literature supports the efficacy of protein- and amino acid-based supplements in improving skeletal muscle mass index, handgrip strength, gait speed, and overall physical performance scores among older adults with or at risk of sarcopenia [23,24]. Importantly, these interventions are generally well tolerated, with reported adverse events being infrequent and typically mild.

Figure 1 shows the reasons for supplement use according to sarcopenia risk. Cognitive decline was the most common reason in both groups and was significantly more frequent in patients at risk (Yes 22/92 vs. No 16/70; *p* = 0.020). Other reasons—such as asthenia, electrolyte imbalance, hair loss, iron deficiency, and visual impairment—did not differ significantly between groups (all *p* > 0.05). Most supplements were prescribed by specialists (83.7%), reported benefits were positive in two-thirds of patients (66.3%), and side effects occurred in only 4.3%. Cognitive decline emerges as the most common reason in both groups, with slightly higher frequency among those not at risk, indicating a multifaceted association between supplements and mental health, with certain of them demonstrating encouraging effects [25]. Other prevalent reasons include electrolyte imbalance, hair loss, and hearing loss, though the distribution varies between groups. Notably, iron deficiency appears more frequent among individuals at risk of sarcopenia, while hearing loss is more common in those not at risk. Overall, the results indicate that although some reasons for taking supplements are common, health concerns vary depending on sarcopenia risk.

The findings of the present study should be considered in the context of both its limitations and its strengths. First, the study was conducted at a single center, which may restrict the external validity of the results and limit their applicability to other settings or populations. Second, supplement use was assessed through self-reported questionnaires, which are inherently subject to recall bias and potential misreporting. Additionally, the absence of detailed data on supplement dosage, duration of use, and adherence precluded a more precise evaluation of exposure and hindered the assessment of possible dose–response relationships. It is also important to underline that another limitation of this study is potential selection bias; recruiting exclusively from cognitive and osteoporosis clinics limits the generalizability of our findings. Patients with osteoporosis are inherently more likely to receive supplement prescriptions (e.g., Calcium/Vitamin D), which likely accounts for the high prevalence of supplement users in our cohort. Finally, due to the cross-sectional design of the study and the lack of data regarding the exact duration of dietary supplement use, we cannot establish a causal relationship or determine the temporal sequence of events. We cannot rule out the presence of reverse causality, wherein a pre-existing decline in physical and muscle function may have prompted the clinical recommendation and subsequent use of supplements. Longitudinal studies are strictly required to disentangle these two distinct scenarios.

Notwithstanding these limitations, the study offers several noteworthy strengths. To our knowledge, it represents one of the first attempts to investigate the association between risk of sarcopenia and the use of dietary supplements in an older outpatient population. Moreover, the study provides an early and comprehensive examination of supplement use among older adults at risk of sarcopenia, contributing novel insights to the adoption of products targeting cognitive health and overall well-being.

In conclusion, dietary supplement use was highly prevalent among older adults at risk of sarcopenia and remained independently associated with sarcopenia risk after adjustment for potential confounders. Individuals at risk showed specific patterns of supplement use, with cognitive decline representing the most common reason for intake. These findings highlight the importance of routinely assessing muscle health in older adults using supplements and support the need for integrated interventions combining targeted nutritional strategies with physical activity. Further longitudinal studies are needed to clarify the direction and clinical implications of this association.

## Figures and Tables

**Table 1:** Descriptive characteristics of patients included.

	Dietary Supplements Non-Users	Dietary Supplements Users	Overall	*p* Values
	(N = 70)	(N = 92)	(N = 162)	
Age				
Mean (SD)	74.6 (8.03)	75.1 (6.68)	74.9 (7.28)	0.67
Risk of sarcopenia (SARC-F ≥ 4)				
No	43 (61.4%)	41 (44.6%)	84 (51.9%)	**0.04**
Yes	27 (38.6%)	51 (55.4%)	78 (48.1%)	
SARC-F: Strength				
Median (IQR)	1 (0–1)	1 (0–2)	1 (0–2)	0.25
SARC-F: Assistance in walking				
Median (IQR)	0 (0–1)	1 (0–2)	0 (0–1)	**0.004**
SARC-F: Rise from a chair				
Median (IQR)	0 (0–1)	1 (0–1)	0 (0–1)	**0.04**
SARC-F: Climb stairs				
Median (IQR)	1 (0–1)	1 (0–2)	1 (0–2)	0.18
SARC-F: Number of falls				
Median (IQR)	0 (0–1)	0 (0–1)	0 (0–1)	0.75
Sex				
F	46 (65.7%)	54 (58.7%)	100 (61.7%)	0.45
M	24 (34.3%)	38 (41.3%)	62 (38.3%)	
Marital status				
Single	22 (31.4%)	24 (26.1%)	46 (28.4%)	0.73
Married	43 (61.4%)	60 (65.2%)	103 (63.6%)	
Widowed	5 (7.1%)	8 (8.7%)	13 (8.0%)	
Educational level				
High School Diploma/University	13 (18.6%)	19 (20.7%)	32 (19.8%)	0.76
Primary/Middle School	52 (74.3%)	64 (69.6%)	116 (71.6%)	
No Formal Education	5 (7.1%)	9 (9.8%)	14 (8.6%)	
Residence				
Rural area	14 (20.0%)	14 (15.2%)	28 (17.3%)	0.557
Urban area	56 (80.0%)	78 (84.8%)	134 (82.7%)	
Smoking				
Former smoker	22 (31.4%)	32 (34.8%)	54 (33.3%)	0.47
Yes	15 (21.4%)	13 (14.1%)	28 (17.3%)	
No	33 (47.1%)	47 (51.1%)	80 (49.4%)	
Alcohol				
No	56 (80.0%)	78 (84.8%)	134 (82.7%)	0.55
Yes	14 (20.0%)	14 (15.2%)	28 (17.3%)	
Reported healthy habits				
No	33 (47.1%)	40 (43.5%)	73 (45.1%)	0.76
Yes	37 (52.9%)	52 (56.5%)	89 (54.9%)	
Category of unhealthy habits				
Unhealthy diet	21 (63.6%)	29 (72.5%)	50 (69%)	0.57
Sleep disorders	12 (36.4%)	11 (27.5%)	23 (31%)	
Side effects				
No	/	88 (95.7%)	88 (54.3%)	
Yes	/	4 (4.3%)	4 (2.5%)	
Benefits				
No	/	31 (33.7%)	31 (33.7%)	
Yes	/	61 (66.3%)	61 (66.3%)	
Suggested by				
Self-Medication	/	6 (6.5%)	6 (6.5%)	
GP	/	9 (9.8%)	9 (9.8%)	
Specialist	/	77 (83.7%)	77 (83.7%)	
GP informed				
No	/	8 (8.7%)	8 (8.7%)	
Yes	/	84 (91.3%)	84 (91.3%)	
MNA categories				
Malnourished	31 (44.3%)	41 (44.6%)	72 (44.4%)	0.71
At risk of malnutrition	18 (25.7%)	28 (30.4%)	46 (28.4%)	
Normal nutritional status	21 (30.0%)	23 (25.0%)	44 (27.2%)	

Abbreviation: GP—General Practitioner; MNA—Mini Nutritional Assessment., SD Standard Deviation, IQR Inter Quartile Range.

**Table 2:** Association between sarcopenia risk (SARC-F ≥ 4) and supplement use.

Variable	OR	CI 95%	*p*-Value	OR	CI 95%	*p*-Value
	Model 1			Model 2 *		
No risk of sarcopenia	1 [reference]		1 [reference]	1 [reference]		1 [reference]
Risk of sarcopenia	1.98	1.05–3.76	**0.034**	2.35	1.21–4.72	**0.013**

* adjusted for sex, age, study, marital status, educational level, smoking.

## Data Availability

The original contributions presented in this study are included in the article/Supplementary Materials. Further inquiries can be directed to the corresponding author(s).

## Supplementary Materials

The following supporting information can be downloaded at: https://www.mdpi.com/article/10.3390/geriatrics11040092/s1, Supplementary Table S1: Questionnaire on supplements use and consumption; Supplementary Tabe S2: SARC-F Questionnaire.
