# Supplementary material for: Muscle Matters: Dietary Supplement Use and Sarcopenia Risk
Source: Geriatrics (Basel). 2026 Jul 21;11(4):92. doi: 10.3390/geriatrics11040092 (PMC13397953; doi:10.3390/geriatrics11040092)
Supplement: Supplementary file 1 [file geriatrics-11-00092-s001.zip › geriatrics-4240631-supplementary.pdf]

Supplementary Table S1. Questionnaire on supplements use and consumption.

**1. Age**
______

**2. Gender**
• Male
• Female

**3. Residence**
• Urban area
• Rural area

**4. Marital status**
• Single
• Married
• Separated / Widowed

**5. Education level**
• No formal education
• Primary/Middle School
• High school diploma/ University degree

**6. Smoking**
• No
• Yes
• Former smoker

**7. Alcohol consumption**
• No
• Yes

**8. Do you consider your lifestyle healthy (diet, sleep hours, etc.)?**
• Yes
• No (specify) ________________________________

**9. Do you use food supplements and/or herbal products?**
• No

- Yes

10. Explain the reason

- Arterial hypertension
- Diabetes mellitus
- Dyslipidemia
- Weight loss
- Fatty liver disease
- Varicose veins
- Menopause
- Psychiatric disorders
- Anxiety and/or sleep disorders
- Urological diseases (cystitis, prostate hypertrophy, etc.)
- Headache
- Constipation and/or dyspepsia
- Electrolyte imbalance (Sodium, Potassium, Magnesium, etc.)
- Osteoporosis
- Joint or bone pain
- Flu-like syndromes
- Immune system support
- Pregnancy
- Fertility enhancement
- Sports products (anabolic or other)
- Well-being / personal wellness products
- Products to improve sexual performance
- Other (briefly specify the reason) ________________________________

11. Please indicate the name of the product:

____

12. Do you think you have benefited from taking these products?

- No

- Yes

13. Who recommended them to you?

- General Practitioner
- Specialist Doctor
- Self-medication

15. Is your General Practitioner aware of it?

- Yes
- No

Supplementary Table S2. SARC-F questionnaire

**Component**	**Questions**	**Scoring**
**1. Strength**	How much difficulty do you have in lifting and carrying 10 pounds (≈ 4.5 kg)?	0 = None<br>1 = Some<br>2 = A lot or unable
**2. Assistance in walking**	How much difficulty do you have walking across a room?	0 = None<br>1 = Some<br>2 = A lot, use aids, or unable
**3. Rise from a chair**	How much difficulty do you have transferring from a chair or bed?	0 = None<br>1 = Some<br>2 = A lot or unable without help
**4. Climb stairs**	How much difficulty do you have climbing a flight of 10 stairs?	0 = None<br>1 = Some<br>2 = A lot or unable
**5. Falls**	How many times have you fallen in the past year?	0 = None<br>1 = 1–3 falls<br>2 = 4 or more falls
